# Cutaneous Sinus Tract in Association with Traumatic Injury to the Teeth

**DOI:** 10.5005/jp-journals-10005-1220

**Published:** 2013-10-14

**Authors:** Rahul Mishra, Tayyeb Sultan Khan

**Affiliations:** Senior Lecturer, Department of Pedodontics and Preventive Dentistry Purvanchal Institute of Dental Sciences, Gorakhpur, Uttar Pradesh India; Senior Lecturer, Department of Oral and Maxillofacial Surgery Purvanchal Institute of Dental Sciences, Gorakhpur, Uttar Pradesh India

**Keywords:** Odontogenic infections, Cutaneous sinus tract, Surgical endodontics

## Abstract

The present case report is of trauma episode of the lower anterior teeth, causing pulpal necrosis with periradicular periodontitis, resulting in the occurrence of cutaneous sinus tracts (fistula). Previous misdiagnosis and inappropriate medical treatment were ineffective. Only when properly referred to dentists, the differential diagnosis was made. The guideline to diagnose cutaneous sinus tracts (fistula) is based mainly on accurate pulp sensitivity tests of the involved traumatized teeth. Intraoral and dental examinations are critical in making the diagnosis. The case presented here shows that cutaneous odontogenic sinus tracts associated in traumatized teeth, even in absence of caries or tooth fracture.

**How to cite this article:** Mishra R, Khan TS. Cutaneous Sinus Tract in Association with Traumatic Injury to the Teeth . Int J Clin Pediatr Dent 2013;6(3):205-207.

## INTRODUCTION

Although cutaneous sinus tracts of dental origin have been well documented in the medical and dental literature, these lesions continue to be a challenging diagnosis. The discharge of purulent exudates usually is associated with periapical radiolucent area and goes through tissues and structures along the path of least resistance.^1^ A review of several reported cases reveal that patients have had multiple surgical excisions, radiotherapy, multiple biopsies, and multiple antibiotic regimens, all of which have failed, with recurrence of the cutaneous sinus tract, because the primary dental etiology was never correctly diagnosed or addressed. This report involves a case of cutaneous facial sinus tract of dental origin, its diagnosis and treatment.

## CASE REPORT

**Fig. 1:**
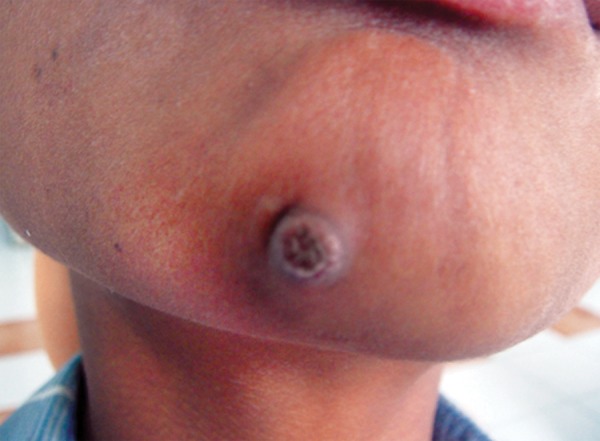
Extraoral photograph

**Fig. 2:**
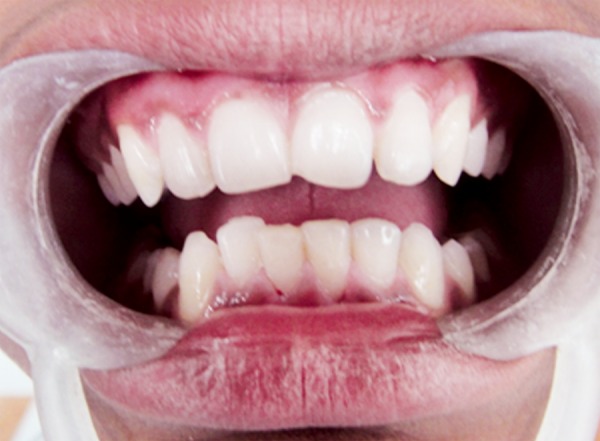
Intraoral photograph

**Fig. 3:**
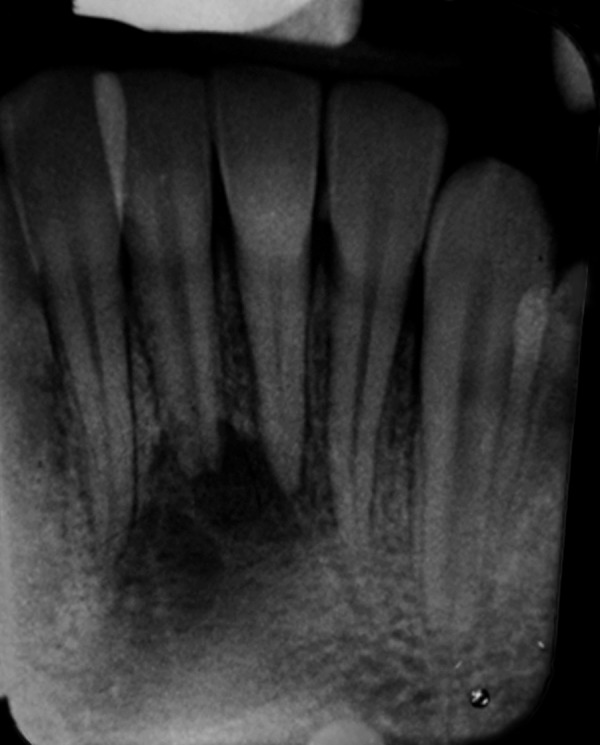
Preoperative radiograph

**Fig. 4:**
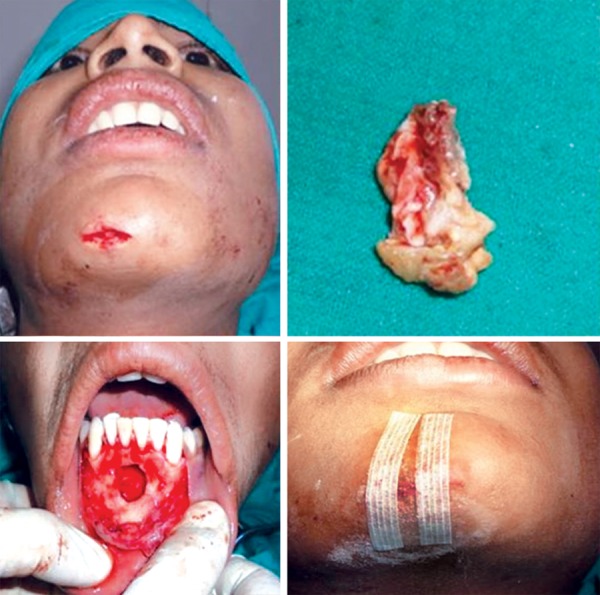
Intraoperative photograph

**Fig. 5:**
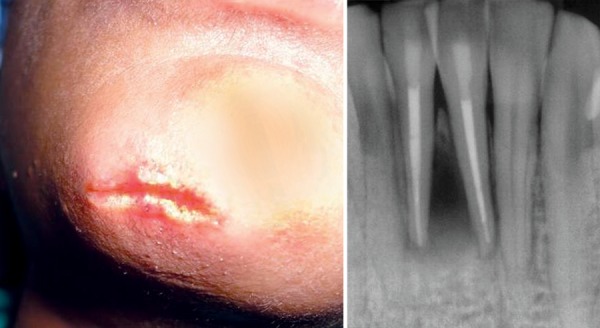
Postoperative photograph and radiograph after 1 month

A 12-year-old boy reported to the Department of Pedodontics and Preventive Dentistry, Babu Banarasi Das College of Dental Sciences, Lucknow with a complaint of episodic drainage from a cutaneous lesion in the mental region of lower jaw. He stated that he had felt an induration about 1 year ago and left it untreated because he had no pain. However, as the lesion started to discharge pus during the following months, he then received several treatments from a dermatologist. Inspite of taking large number of antibiotics and antifungal medications both orally and topically, the lesion did not heal then patient was referred to our college. The patient had also reported a history of trauma to that area 4 years back. Extraoral examination showed a nontender elevated crusty nodule approximately 1 cm in diameter in the mental area (Fig. 1) Palpation elicited an exudatous discharge from it. Intraorally, no vestibular swelling was present. The mandibular incisors had no mobility, and responded within normal limit to percussion, with no detectable periodontal pocket (Fig. 2) A periapical radiograph showed a diffuse radiolucency surrounding mandibular right and left central incisors. Furthermore, patient's age was 12 years with a history of trauma 4 years back but the root apex almost completed which could be due to slow pulpal degeneration (Fig. 3) Vitality tests were performed on all mandibular anterior incisors. The mandibular right and left central incisors did not respond to thermal and electric pulp test. A diagnosis of suppurative apical periodontitis was made on the offending teeth. A surgical excision of extraoral sinus tract was done followed by root canal treatment of involved teeth with antibiotic coverage (Figs 4 and 5).

## DISCUSSION

Cutaneous sinus tract and fistulization of the facial skin have a wide range of etiologies, the most common being odontogenic in origin. Such patients usually seek help from surgeons or dermatologists rather than dentists and often undergo multiple inappropriate treatments. In diagnosis of cutaneous dental fistula, although the examiner usually looks for dental caries or periodontal diseases, he should bear in mind the possibility of dental traumatic injuries.^2-4^ The discharge of purulent exudates usually is associated with periapical radiolucent area and goes through tissues and structures along the path of least resistance.^5^ The site of drainage can be located intra- or extraorally, depending on certain circumstances such as: the tooth which is diseased, and the apex position relatively to muscular attachments, bacterial virulence and lower host resistance.^6^ In a cohort study of 108 odontogenic sinus tracts Slutzky-Goldberg et al.^7^found just 1case with cutaneous sinus tract. In the report of Gupta and Hasselgren,^4^ all odontogenic sinus tracts (29 cases) had intraoral openings. Studies revealed that the extraoral sinus tracts is most commonly found on the cheek, chin and angle of the mandibule, and in this way making the diagnosis more difficult to the clinician. These authors have reported how important is the interaction between physicians and dentists is to avoid submitting patients to multiple biopsies, antibiotic regimens and unnecessary surgery, before correct diagnosis and endodontic therapy are in course.^4,8,9^ The cutaneous sinus tracts are a sequel to pathosis and that the clinician should be able to recognize the primary cause. Therefore, taking the patient's history becames crucial in order to avoid misdiagnosing a wide variety of diseases like ingrow hair, osteomyelitis, local skin infection and neoplasm.^10^ In several cases of teeth traumatism the pulp can be affected, even if the crown-root integrity is not damaged. Odontogenic sinus tracts appear as a papule or nodule with purulent discharge usually in the chin or jaw.^11^The histology of these tracts is often characterized as fragments of granulation tissue that are focally lined by epithelium.^1^ Most infections are polymicrobial, and culture often yields growth of anaerobes or facultative anaerobes, such as *Streptococcus* species. It has been observed that systemic antibiotic therapy will result in a temporary reduction of the drainage and apparent healing. Surgical excision of cutaneous sinus tracts followed by root canal treatment of the affected teeth is the treatment of choice.

## CONCLUSION

Chronic, draining dental infection is one of the most common causes of fistulae of the face and neck. An understanding of the pathogenesis of cutaneous fistulae arising from dental infections will lead to proper early diagnosis and treatment without unnecessary surgery. The case presented here shows that cutaneous odontogenic sinus tracts associated in traumatized teeth, even in absence of caries or tooth fracture; the clinician must investigate the pulpal health of the teeth in the contiguous area of cutaneous fistula. This case report highlights the need for thorough diagnostic procedures that should always include a dental examination. The clinician should recognize that a cutaneous sinus tract is a sequel to pathosis, while the associated nonvital tooth, with its periradicular periodontitis, is the primary cause.
